# Nr4a1-eGFP Is a Marker of Striosome-Matrix Architecture, Development and Activity in the Extended Striatum

**DOI:** 10.1371/journal.pone.0016619

**Published:** 2011-01-28

**Authors:** Margaret I. Davis, Henry L. Puhl

**Affiliations:** 1 Laboratory for Integrative Neuroscience, National Institute on Alcohol Abuse and Alcoholism, National Institutes of Health, Bethesda, Maryland, United States of America; 2 Laboratory of Molecular Physiology, National Institute on Alcohol Abuse and Alcoholism, National Institutes of Health, Bethesda, Maryland, United States of America; Biological Research Center of the Hungarian Academy of Sciences, Hungary

## Abstract

Transgenic mice expressing eGFP under population specific promoters are widely used in neuroscience to identify specific subsets of neurons in situ and as sensors of neuronal activity in vivo. Mice expressing eGFP from a bacterial artificial chromosome under the Nr4a1 promoter have high expression within the basal ganglia, particularly within the striosome compartments and striatal-like regions of the extended amygdala (bed nucleus of the stria terminalis, striatal fundus, central amygdaloid nucleus and intercalated cells). Grossly, eGFP expression is inverse to the matrix marker calbindin 28K and overlaps with mu-opioid receptor immunoreactivity in the striatum. This pattern of expression is similar to Drd1, but not Drd2, dopamine receptor driven eGFP expression in structures targeted by medium spiny neuron afferents. Striosomal expression is strong developmentally where Nr4a1-eGFP expression overlaps with Drd1, TrkB, tyrosine hydroxylase and phospho-ERK, but not phospho-CREB, immunoreactivity in “dopamine islands”. Exposure of adolescent mice to methylphenidate resulted in an increase in eGFP in both compartments in the dorsolateral striatum but eGFP expression remained brighter in the striosomes. To address the role of activity in Nr4a1-eGFP expression, primary striatal cultures were prepared from neonatal mice and treated with forskolin, BDNF, SKF-83822 or high extracellular potassium and eGFP was measured fluorometrically in lysates. eGFP was induced in both neurons and contaminating glia in response to forskolin but SKF-83822, brain derived neurotrophic factor and depolarization increased eGFP in neuronal-like cells selectively. High levels of eGFP were primarily associated with Drd1+ neurons in vitro detected by immunofluorescence; however ∼15% of the brightly expressing cells contained punctate met-enkephalin immunoreactivity. The Nr4a1-GFP mouse strain will be a useful model for examining the connectivity, physiology, activity and development of the striosome system.

## Introduction

Principle neurons in the telencephalon are organized into layers with distinct circuit and ensemble functions that can be surmised simply based on the location of the nuclei but the anatomical organization of the striatum has proven more challenging. This is because the striatum does not possess the readily identified laminar organization of most telencephalic structures and because the majority of striatal neurons are of one class, the GABAergic Medium Spiny Neuron (MSN) [1]. Recent studies have made use of the major distinction in MSN classes, differential expression of dopamine receptors (Drd1 or Drd2-GFP) in putative direct and indirect pathway neurons [2], respectively, to examine differential plasticity in the striatum but this technique only addresses one level of striatal complexity. The striatum is grossly divided into Dorsolateral (DLS), Dorsomedial (DMS) and ventral/Nucleus Accumbens (NAc). These divisions are roughly equivalent to motor, associative and limbic subdivisions but exist more as a dorsolateral to ventromedial gradient [3]. Regions can readily be identified for gross analysis but there is yet another layer of afferent-efferent and neurochemical heterogeneity within the striatum, the striosome-matrix organization.

Little is known about the differential function of the striosomes compared with the surrounding matrix. Existing data indicate that the dorsolateral matrix primarily serves motor functions [4] while partial ablation of dorsal striosomes impairs rotorod learning [5], suggesting cross talk between these regions during skill acquisition. Striosomes are a preferred striatal region for self-stimulation with implanted electrodes [6] and receive preferential and regionally selective innervation from the basolateral amygdala, prelimbic, infralimbic, orbitofrontal and anterior cingulate cortices and project primarily to the substantia nigra pars compacta (SNpc; reviewed in [7], [8], [9]). This is in contrast to dorsolateral matrix neurons, which receive innervation from sensorimotor cortex [4], [10], [11]. Matrix neurons are high in enkephalin, a marker of indirect pathway neurons, and project to the external segment of the globus pallidus (GPe) [2],[7]. The matrix neurons are also the main target of vesicular glutamate transporter type two-containing thalamostriatal efferents [12]. Striosomal neurons contain substance P and dynorphin, markers of direct pathway neurons [2], [13], [14], [15], [16]. Direct pathway neurons should therefore concentrate in the striosomes while indirect pathway neurons should concentrate in the matrix. This simple division has not been supported with data using Drd1 or Drd2-driven eGFP, however, and is further complicated by the observation that a 5-17% of striatal neurons co-express Drd1 and Drd2 depending on the region [2]. Combined, these data indicate that striatal organization and function is far more complex than either direct-indirect or striosome-matrix dichotomies imply.

Much of what is known about striosome-matrix organization and development has relied on catecholamine fluorescence [17], [18], receptor binding [17], [19], [20], [21] and immunohistochemistry [22], [23], [24] but eGFP expression has recently been used to address differential development and plasticity in the two compartments. The proenkephalin-eGFP mouse has preferential matrix expression [25] and the early innervation by dopaminergic fibers using the Tyrosine Hydroxylase (TH)-eGFP mouse has been exploited to identify striosomes developmentally [26]. Fluorescent protein expression offers a static view based on differential transcriptional activity between neuronal populations but eGFP expression can also be used as a sensor of neuronal ensemble and immediate-early promoter activity [27]. Identification of a reporter line with differential expression in the striosome and matrix that is also a reporter of pathway/ensemble plasticity and activity would greatly expand our ability to address more sophisticated questions about striosome-matrix physiology in vivo and in situ.

The GENSAT database (www.gensat.org) was screened for images with differential striosome vs. matrix expression and we identified the Nr4a1 (Nuclear receptor subfamily 4, group A, member 1) line as a candidate activity-dependent reporter. The Nr4a1 gene (Entrez Gene ID: 15370, http://www.ncbi.nlm.nih.gov/gene/15370) is also known as GFRP1, Gfrp, Hbr-1, Hbr1, Hmr, N10, NGFI-B, NGFIB, NP10, TIS1, TR3 and Nur77. Nr4a1 is a widely expressed orphan of the steroid-thyroid hormone receptor superfamily that is readily inducible at the level of transcription. Nuclear Nr4a1 monomers bind the NGFI-B response element while homodimers bind the Nur77 response element and Nr4a1 heterodimerizes with the retinoic acid receptor to bind the DR5 motif (reviewed by [28]). The Nr4a1 promoter contains consensus sites for multiple transcription regulators and is induced by calcium, protein kinase C, mitogen-activated protein kinases and cAMP dependent transactivation depending upon the cell type [29], [30], [31], [32], [33] while Nr4a1 protein is subject to extensive post-transcriptional regulation [34], [35], [36]. Nr4a1 induction parallels other immediate early genes within the basal ganglia. Nr4a1 mRNA [37], but not protein [38], is enriched in direct pathway neurons. Nr4a1 is induced by psychostimulant exposure [39], [40], [41] and during opiate withdrawal [42], [43]. Antipsychotics also induce Nr4a1 expression throughout the basal ganglia, although the pattern and level of induction vary with drug subclass and even among members of the same pharmacological class [44]. Together, these data implicate the Nr4a1 gene in plasticity in the basal ganglia that occurs during psychostimulant and antipsychotic exposure and suggest that the pattern of induction may be reflective of differential pharmacological effects within the circuit.

A reporter mouse strain with inducible expression would therefore be useful for identifying circuits involved in short and long term plasticity after stimulant and antipsychotic exposure but also with striatal-based learning paradigms. Here we report the characterization of striosome-matrix and activity-dependent expression of eGFP from the Nr4A1 promoter as both an anatomical marker for striosomes and a reporter for activity in the extended striatum. Expression occurs primarily in Drd1+ neurons but the level of basal and stimulated expression varies with compartment, thereby differentiating Drd1 striosome neurons from Drd1 matrix neurons. These mice will be useful for examination of drug- or activity-dependent Nr4a1 induction in the extended striatum associated with learning and plasticity as well as for in situ identification of neurons differentially activated within the striosome and matrix compartments.

## Results

### Distribution of Nr4a1-eGFP in the Mature Basal Ganglia

**Figure 1:**
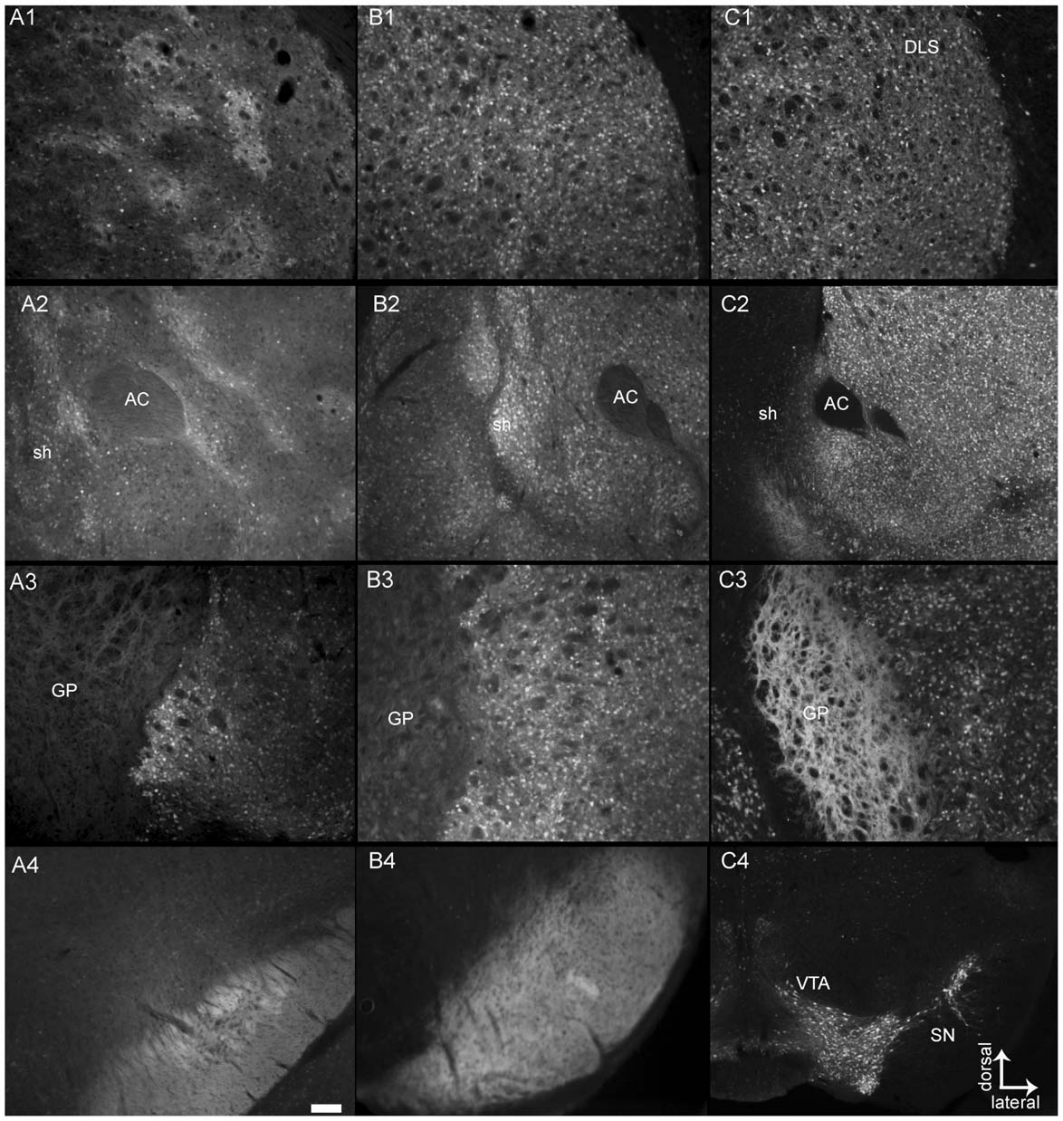
Nr4a1-eGFP expression in the basal ganglia. Nr4a1-eGFP expression (A1–A4) in the striatum and striatal projection areas compared to Drd1-eGFP (B1–B4) and Drd2-eGFP (C1–C4) expression in the mature mouse. The DLS is shown in row 1, ventral striatum in row 2, globus pallidus in row 3 and SN/VTA in row 4. The scale bar, 200 µm, applies to all images.

Nr4a1 promoter driven eGFP expression was observed in distinct cell populations throughout the brain and periphery (Figs. S1 and S2). eGFP levels were particularly high in the mature striatum but the pattern was distinct from both Drd1-eGFP and Drd2-eGFP expression (Fig. 1). Expression in the dorsal striatum was striosome-like in the Nr4a1 strain (Fig. 1 A1) but uniform in both the Drd1 (Fig. 1 B1) and Drd2 (Fig. 1 C1) strains. The expression pattern is in agreement with previous reports using the Drd1 and Drd2 strains [2]. Nr4a1-eGFP was laminar in the ventromedial striatum and patch/swirl-like in the shell of the NAc (Fig. 1 A2). A similar heterogeneous expression pattern was apparent in the Drd1-eGFP strain (Fig. 1 B2) although these regions of intense expression in the NAc shell covered a larger area in the Drd1-eGFP strain. Drd2-eGFP expression was more uniform in the ventral striatum and NAc (Fig. 1 C2) but, unlike the Nr4a1 and Drd1 strains, was low in the NAc shell. Expression within fibers in the GP was low in both the Nr4a1 (Fig. 1 A3) and Drd1 (Fig. 1 B3) strains but robust in the Drd2 strain (Fig. 1 C3). Expression was also detected in fibers in the SN of both the Nr4a1 (Fig. 1 A4) and Drd1 (Fig. 1 B4) strains. Regions of patchy innervation were sometimes observed in the SNpc of the Nr4a1 strain (Fig. 1 A4). In contrast, expression in the ventral mesencephalon of the Drd2 strain was mainly restricted to somata within the SN and Ventral Tegmental Area (VTA; Fig. 1 C4). This pattern of expression is consistent with Nr4a1 expression being primarily in direct pathway neurons but is not identical to Drd1-eGFP expression. Direct pathway neurons project primarily to the SN, however, collaterals also innervate the GP [45], [46], therefore expression in this region may be eGFP in these collaterals, fibers en passant or could represent a subpopulation of indirect pathway neurons that express Nr4a1-eGFP [39]. The differential intensity of the innervation of the SNpc may represent the terminals of striosome neurons, which are known to project preferentially to the SNpc and have segmented striosome-matrix projections [7], [9].

**Figure 2:**
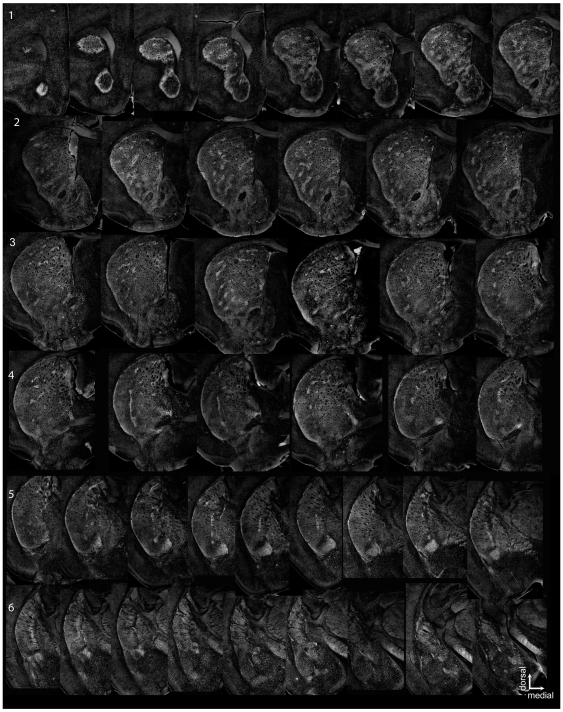
Serial sections through the striatum and extended amygdala of a mature Nr4a1-eGFP mouse. Striosomes appear brighter than the surrounding matrix, particularly the subcallosal streak (rows 1–2) and lateral striatal streak (rows 3–5), while a laminar organization is apparent in the ventromedial striatum (rows 1–4). Regions of intense expression are also present within the BNST (rows 4 and 5), the striatal fundus (IPAC, row 5) and within the intercalated cells of the amygdala (row 6). A uniform background level was subtracted from these images using Image J. When sections were missing or damaged, adjacent or contralateral sections were substituted. Intervals - range from 80–120 µm. Due to differences in mouse strain of the reference atlas (Allen Brain Atlas, C57/Bl6J) and tissue shrinkage the coordinates are approximate.

Serial sections through the striatum and amygdala (Fig. 2) revealed striosomes that form near-contiguous layers. The subcallosal streak and lateral striatal streak have contiguous expression, framing the striatum (Fig. 2 rows 1, 2). These cells were similar in intensity and proximity to fiber tracts as cells of the lateral and medial Intercalated Cells (ITC) of the amygdala (Fig. 2 row 6). Apparent layers in the striatum are often interrupted by blood vessels and fiber bundles, particularly in dorsomedial regions, but resemble the laminar organization previously demonstrated in a serial reconstruction [47]. Rows in the matrix have been identified in anterograde tracer studies from motor cortex [11] but these lamina also resemble striosomes innervation from deep layer prefrontal cortex developmentally and in the adult [48], indicating that the projections to the striosome and matrix may be arranged in parallel. Striosomes were also frequently observed in close proximity to and surrounding large blood vessels, as has previously been noted [49]. Layers were curved parallel to the corpus callosum in the dorsal and lateral regions of the rostral striatum but formed dorsomedial to ventrolateral layers in the ventral striatum (Fig. 2 rows 2, 3). Nr4a1-eGFP expression in the NAc, Bed Nucleus of the Stria Terminalis (BNST) and amygdala was high and variable between animals but was never uniform (Fig. 2 rows 4–6). High expression was evident in the striatal fundus (Interstitial Nucleus of the Posterior limb of the Anterior Commissure, IPAC, Fig. 2 row 5) that continued into the ITC of the amygdala and the Central nucleus of the Amygdala (CeA). The CeA contained numerous intensely fluorescent MSN-like cells. eGFP expression within the lateral amygdala and basolateral amygdala was sparse with low level expression in large neurons.

**Figure 3:**
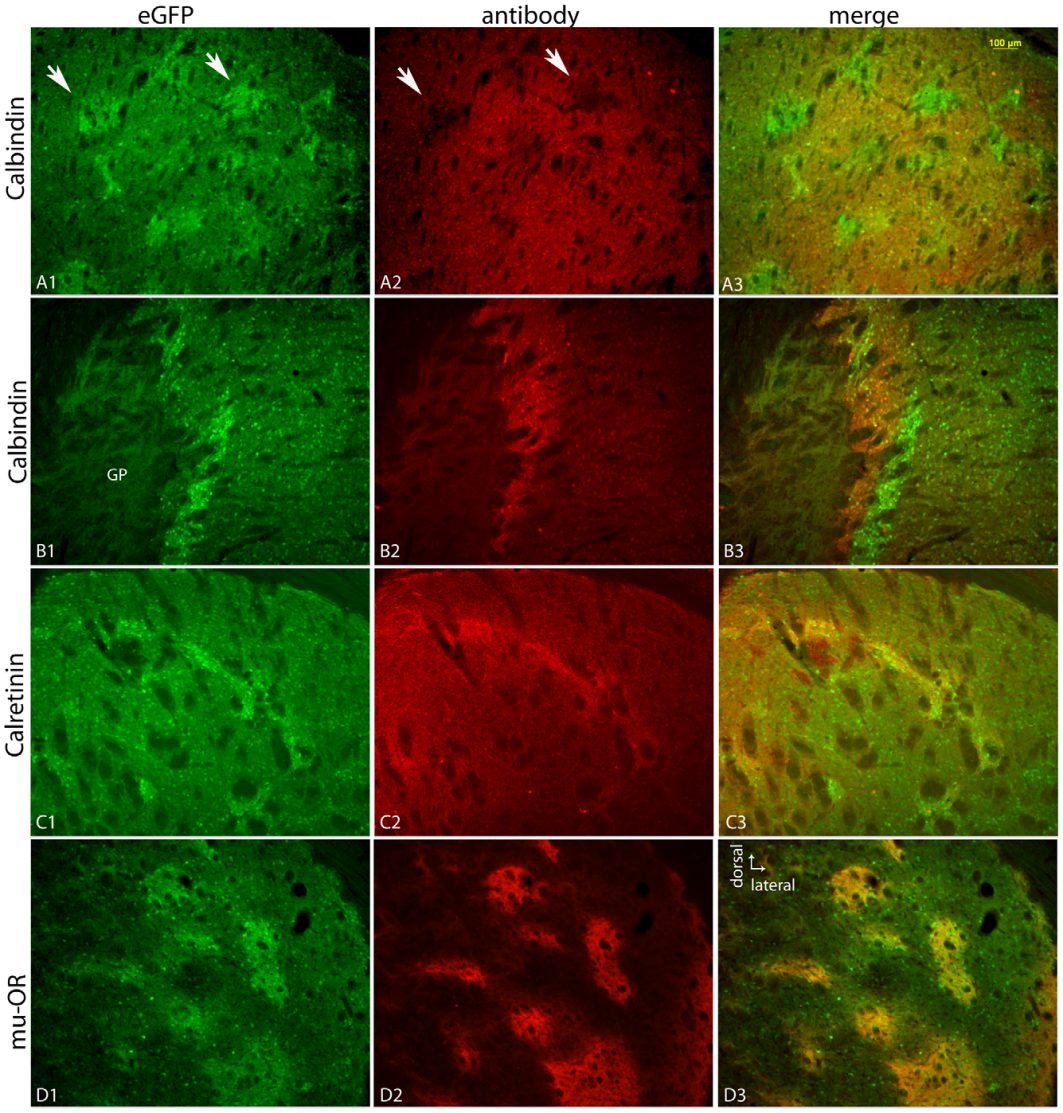
Nr4a1-eGFP expression in the striatum compared to the matrix marker calbindin 28K and striosome markers calretinin and mu-OR. eGFP is shown in the left column. Calbindin expression in the dorsolateral striatum (A2) and globus pallidus/caudal striatum (B2) is merged with eGFP in A3 and B3. Calretinin immunoreactivity in the dorsomedial striatum is shown in C2 and merged with eGFP in C3. Mu-OR activity (D2) is merged with eGFP in D3. The scale bar in A1 (100 µm) applies to all panels.

Multi-label immunofluorescence was used to verify striosomal expression of eGFP in the Nr4a1 strain (Fig. 3). Calbindin 28K is a marker for matrix neurons in the mature striatum [50], [51], [52] and reciprocal expression occured between Nr4a1-eGFP and the matrix-associated calcium-binding protein calbindin 28K in the striatum (Fig. 3, A1–B3). This was apparent in both rostral (Fig. 3, A1–A3) and caudal regions (Fig. 3, B1–B3). Fluorescence was also apparent in the surrounding matrix cells in the adult mouse brain but at a lower intensity. Calretinin immunoreactive fibers from the paraventricular nucleus of the thalamus were present in striosomes (Fig. 3, C1–C3) in the limbic and associative striatum in a gradient that increased from the dorsolateral to ventromedial direction and along the septal pole [53], [54]. eGFP overlapped with this marker in more dorsomedial regions but eGFP was stronger in the weakly calretinin innervated subcallosal streak while calretinin-immunoreactive fibers were more dense in the medial and ventral striatum (see below). The classical striosomal marker, the mu-OR, also colocalized with Nr4a1-eGFP in the dorsal striatum (Fig 3, D1–D3).

**Figure 4:**
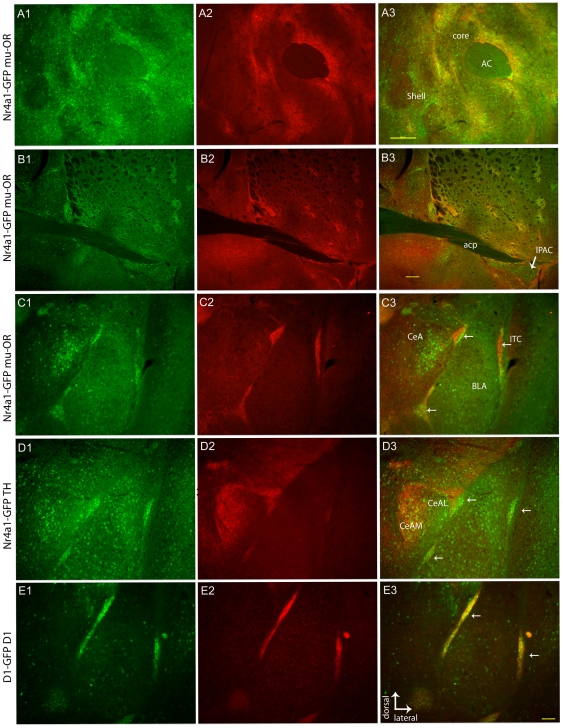
Nr4a1-eGFP expression compared with striosomal markers in the extended striatum and amygdala. Nr4a1-driven eGFP expression is present in the NAc (A1), BNST (B1) and amygdala (C1, D1). Robust colocalization with mu-OR is observed in the NAc core but not the shell (A2, A3). The BNST shows heterogeneous eGFP expression (B1) that overlaps with mu-OR immunoreactivity in most subregions (B2, B3). Mu-OR immunoreactivity in the amygdala (C2) overlaps with Nr4a1-eGFP expression in the ITC (C3, arrows) but not in the CeA. TH immunoreactivity is not uniform with greater innervation of the medial CeA (CeAM) than lateral CeA (CeAL) at this level (D2). TH+ fibers innervating the ITC were faint compared to the medial CeA but overlapped with the ITC (D2, D3). Drd1-eGFP expression (E1, E3 merge) and Drd1 immunoreactivity (E2, E3) in the amygdala are shown for comparison. Scale bars in A3 and B3 are 200 µm. Scale bar in E3 is 100 µm and applies to panels C–E.

Heterogeneous eGFP expression was also observed in the extended striatum, including the NAc, BNST, ITC and the CeA. Images through the extended striatum (Fig. 4) reveal overlap with the mu-OR in the ventral striatum and NAc in laminar and swirled patterns (Fig. 4, A1–A3). “Columns” and “zones” have been used to described these regions based on differential neuropeptide and calcium binding protein expression [53], [55], [56], [57]. Anterograde tracing of deep layer prefrontal afferents identified a similar laminar distribution in the ventral striatum and NAc [48]. Most subregions of the BNST (Fig. 4, B1–B3) contain mu-OR and Nr4a1 colocalization; however, interestingly, the IPAC at the lateral edge and ventral to the posterior commissure does not contain high levels of mu-OR but expresses Nr4a1-eGFP (arrow, Fig. 4 B3).

It has been previously suggested the ITC neurons are striatal in origin [58] and recent studies have confirmed this by mapping lineage-specific transcription factors [59], [60]. Like striosomes, the ITC is rich in mu-OR expression [61] and mu-OR immunoreactivity overlaps with Nr4a1-GFP expression in the ITC (Fig. 4, C1–C3). The ITC is also rich in dopaminergic fibers [62] and TH immunoreactivity overlaps with Nr4a1 expression in the ITC (Fig. 4, D1–D3). This overlap is not as robust in the CeA where Nr4a1 expression is lighter overall but areas of high Nr4a1-GFP expression are observed, particularly in the medial region that receives dense dopaminergic innervation (Fig. 4, D1–D3). For comparison, Drd1-eGFP expression in the CeA and ITC revealed both low Drd1 immunoreactivity and low eGFP levels in the CeA but high expression in the ITC (Fig. 4, E1–E3). While Nr4a1 expression is consistent with the ITC being striosome-like, Drd1 immunoreactivity and Drd1-eGFP expression suggests the CeA has fewer Drd1 and mu-OR expressing neurons and lower Nr4a1 expression under basal conditions than the ITC. Combined, this pattern of immunoreactivity is consistent with the ITC being derived from the lateral ganglionic eminence and phenotypically striosome-like [59].

**Figure 5:**
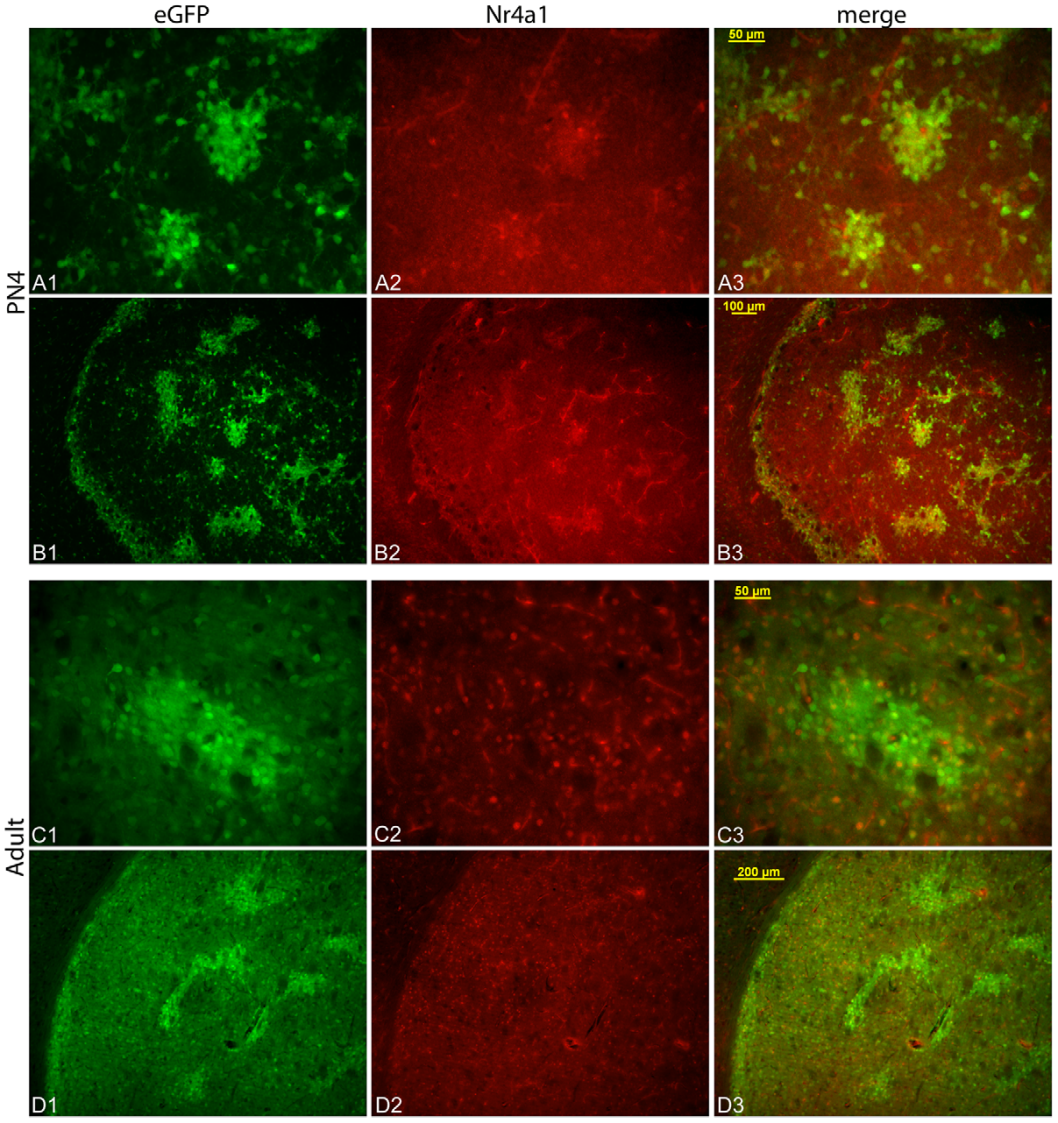
Immunolocalization of endogenous Nr4a1 in the developing and adult striatum. Dorsolateral striatum from a neonatal (PN3/4, A, B) and mature mouse (PN30, C, D). Higher power images indicate colocalization of eGFP and nuclear Nr4a1 in the developing dopamine islands (A1–A3) that is present throughout the structure at low power (B1–B3). In contrast, there is little overlap between nuclear Nr4a1 expression and eGFP in the mature striatum (C, D). High power images detected a speckled distribution (C2) in addition to nuclear localization. The lack of correlation between eGFP levels and Nr4a1 is shown in low power images of the mature dorsolateral striatum (D1–D3). The scale bars in A and C are 50 µm, 100 µm for B and 200 µm for D.

Nr4a1 is extensively regulated at the post-transcriptional level and the protein has a short half-life of 2–4 hrs [36], [63], therefore eGFP levels (half-life ∼26 hrs) may not reflect native protein levels (supplemental text in [64]). Immunostaining for Nr4a1 in neonatal mice indicated extensive overlap in expression of eGFP and endogenous protein levels in the dorsal striatum (Fig. 5 A1-3, B1-3). However, expression diverged in the adult striatum with very little correlation between the endogenous nuclear protein levels and eGFP, particularly in the matrix where Nr4a1 immunoreactivity was detected in eGFP negative cells. Nr4a1 expression was primarily localized to the nucleus in the developing and mature animals (Fig. 5 A2, C2) but a background haze was also present, consistent with descriptions of Nr4a1 mitochondrial localization [34], [35].

### Development of Nr4a1-eGFP Expressing Striosomes

**Figure 6:**
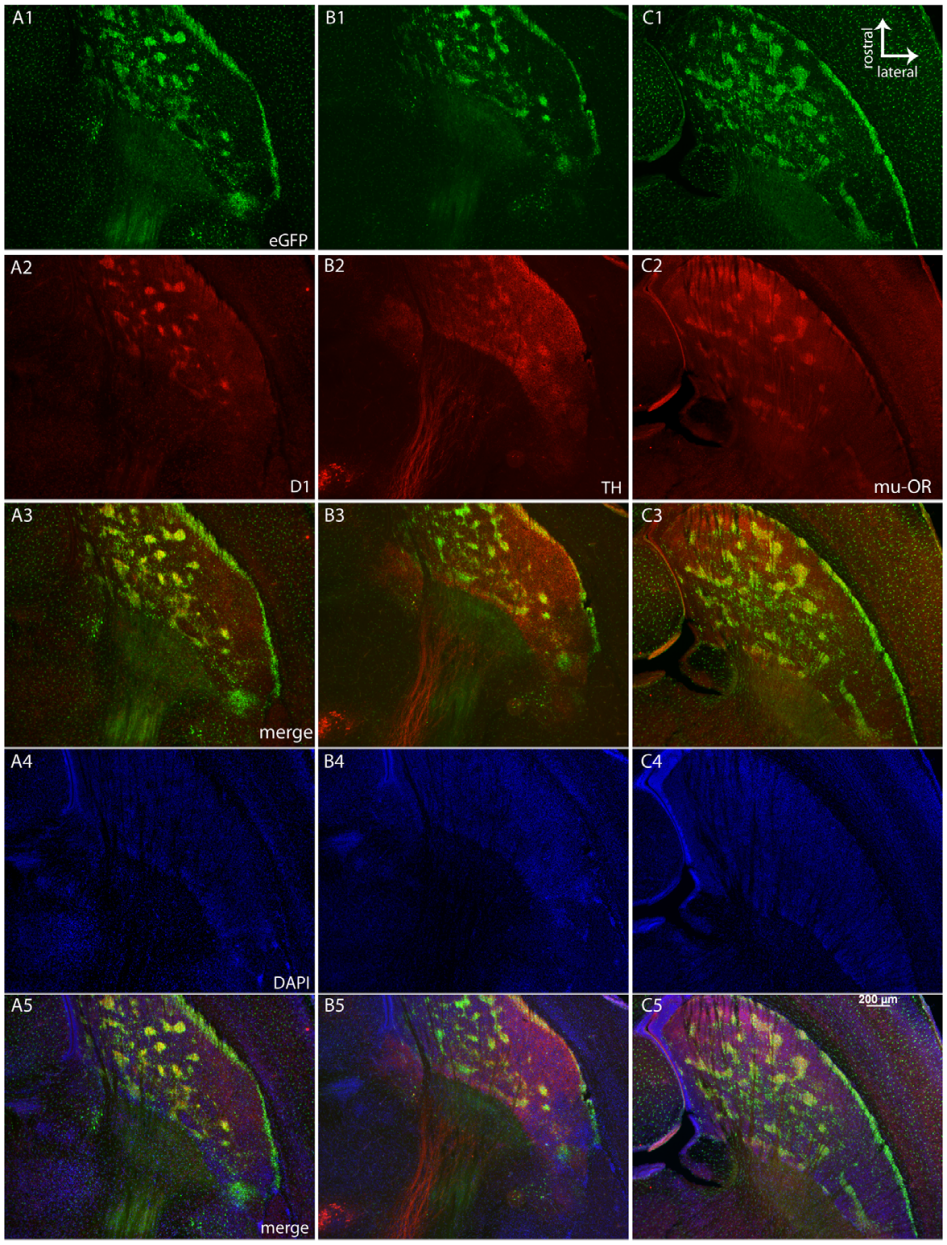
Developmental expression of Nr4a1 in striatonigral projections compared to Drd1, TH and mu-OR in horizontal sections. eGFP expression (A1, B1, C1) overlaps with Drd1 immunoreactivity (A2, A3, A5 merged with the DAPI channel) and TH immunoreactivity (B2, B3, B5 merged with the DAPI channel) Mu-OR immunoreactivity (C2) colocalized with Nr4a1-eGFP is shown in C3 and in C5 merged with the DAPI channel. DAPI staining is less intense in the striosomes (A4, B4, C4), indicating that the striosome-like distribution is not due to increased cell density. The scale bar in H (200 µm) applies to all images. Panels A and B were taken with a Zeiss Axiovert microscope. Panels in C were taken with a Zeiss Lumar stereomicroscope.

Dopaminergic innervation of the developing striatum has been extensively studied. Striosome neurons are born first and occupy “dopamine islands” that are rich in dopamine and TH immunoreactive fibers [18], [65], [66] and the Drd1 dopamine receptor [67], [68]. This pattern of expression extends into the neonatal period [67], [68], [69], [70]. When eGFP was examined in horizontal sections from the newborn (Post Natal, PN 3/4) Nr4a1-eGFP striatum, an island-like expression pattern was observed with very little expression in the developing matrix compartment (Fig. 6 A1, B1, C1). This pattern of expression overlapped with Drd1 immunoreactivity (Fig. 6 A2, A3) and was not due to differences in cell density observed with DAPI binding (Fig. 6 A4, A5). A similar pattern of colocalization was observed with TH immunoreactivity (Fig 6 B1–B3) and DAPI staining is shown for comparison (Fig. 6 B4). Mu-OR staining showed robust overlap in the medial striatum at this age (Fig. 6 C1–C3) but eGFP was stronger in the ventrolateral and caudal regions where mu-OR immunoreactivity was absent (Fig. 6 C3).

**Figure 7:**
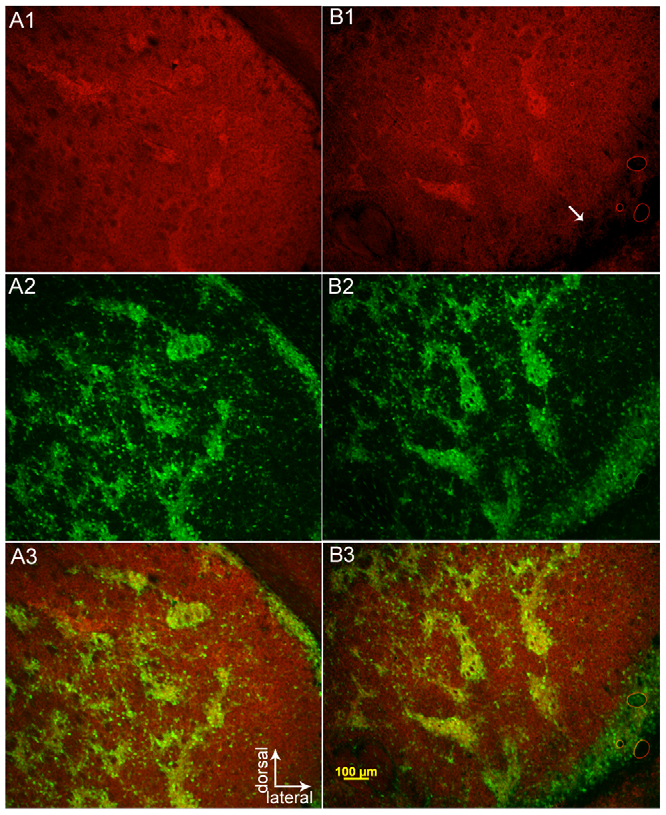
TrkB and Nr4a1 are co-expressed in developing striosomes. TrkB expression (A1, B1) and Nr4a1-eGFP (A2, B2) merged (A3, B3) in the developing striosomes at PN3/4. Dorsolateral (A) and ventrolateral striatum (B) are shown. Arrow (B1) indicates the lateral striatal streak. Scale bar in J (100 µm) applies to all images.

TH immunoreactivity in horizontal sections through the neonatal striatum indicated selective dopaminergic innervation of the developing striosomes and a close association between the developing dopamine system and eGFP expression. Developing striosomes are also rich in TrkB [71] and cortical afferents are the main source of BDNF [72]. Thus, cortical afferents may play a role in striatal maturation. Nr4a1 expression overlaps with TrkB in both dorsal (Fig. 7, A1–A3) and ventral (Fig. 7, B1–B3) striosomes. This was consistent throughout the striatum but not in the subcallosal streak and the lateral striatal streak at PN3/4 (Fig. 7, B1–B3) where Nr4a1-eGFP was expressed at high levels but TrkB immunoreactivity was low. Concordance with these established developmental patterns suggested that Nr4a1-eGFP is closely associated with Drd1 expression and dopaminergic activity in the developing striatum but association with TrkB suggests a role for cortical afferents in Nr4a1-eGFP expression during striosome development. Low expression of TrkB and mu-OR in the ventral/lateral striatal streak suggests that these striosome-like regions may be physiologically or developmentally divergent.

**Figure 8:**
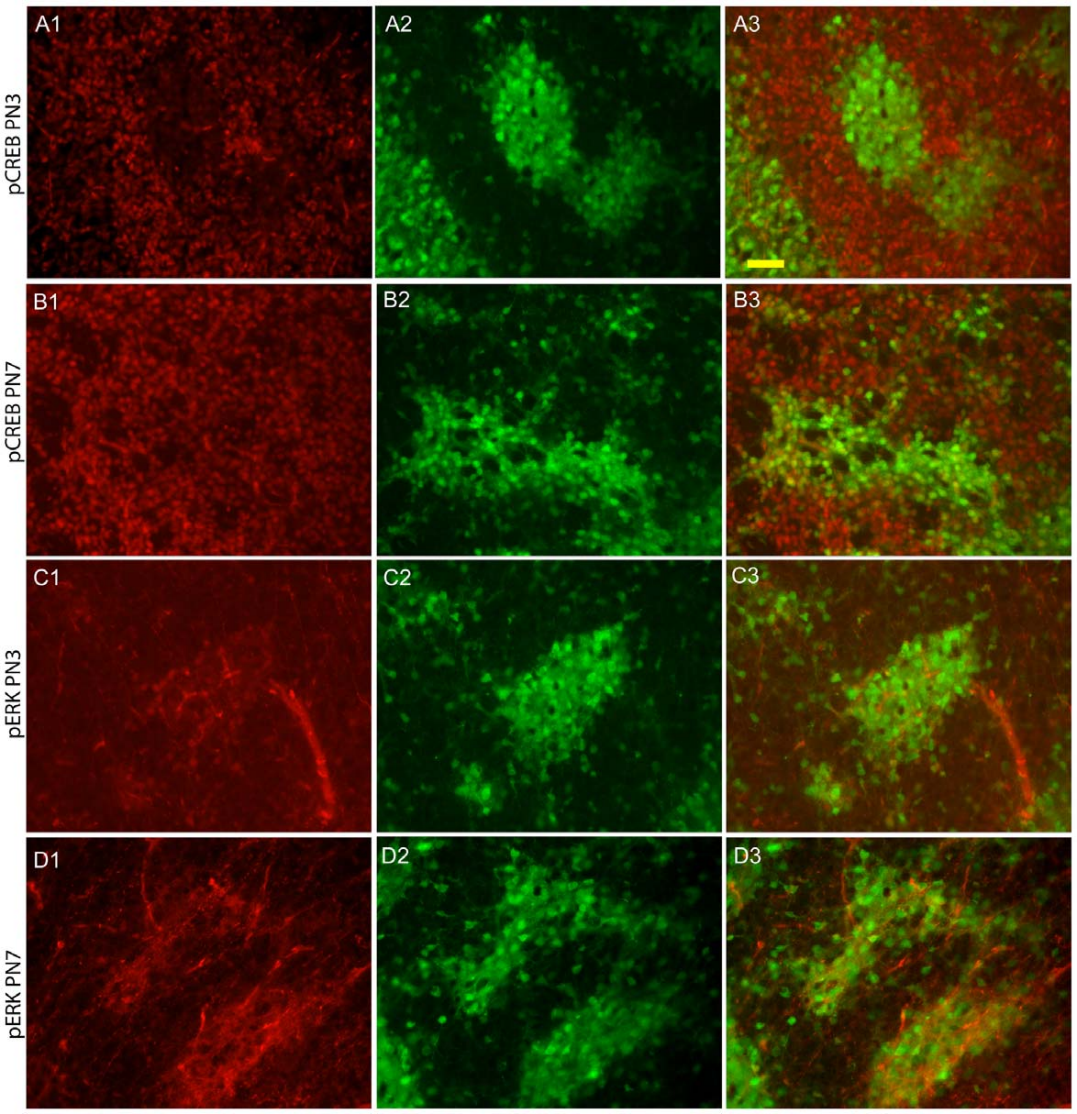
Phosphorylation of CREB and ERK in the neonatal Nr4a1-eGFP striatum. Phospho-CREB immunoreactivity is lower in the striosomes than in the surrounding matrix at PN3/4 (A1–A3) and PN7 (B1–B3). ERK phosphorylation shows the opposite pattern of immunoreactivity at both PN3/4 (C1–C3) but the distribution of pERK changes during development with nuclear and process localization at PN7 (C1 compared to D1). Nr4a1 and active ERK and CREB were also detected in the vasculature. Scale bar in A3 (50 µm) applies to all images.

Nr4a1 induction can be mediated by numerous overlapping signaling pathways but CREB is a common stimulus for activation in multiple systems [28]. We therefore surmised that CREB and/or ERK may be involved due to the overlap with Drd1 and TrkB expression in the developing neonatal striatum. Surprisingly, immunofluorescent detection of Ser 133 phosphorylated CREB (p-CREB) revealed a reciprocal expression pattern relative to Nr4a1-eGFP at PN3/4 (Fig. 8, A1–A3). pCREB was also observed in the adjacent vasculature at PN3/4. By PN7, pCREB immunoreactivity increased in both compartments and overlap could be observed in cells at the edges of the striosomes and in scattered matrix cells (Fig. 8, B1–B3). In contrast, ERK activation overlaps with Nr4a1-driven eGFP expression at both PN3/4 (Fig. 8, C1–C3) and PN7 (Fig. 8, D1–D3), although the dominant pattern of immunoreactivity shifted with time. At PN3/4, pERK was present primarily within striosomal nuclei (Fig. 8, C1) but this shifted to a combined diffuse and nuclear pattern by PN7 (Fig. 8, D1). pCREB is probably not the main transactivating mediator of Nr4a1-eGFP expression in developing striosomal neurons but ERK-mediated pathways are differentially active in striosomes.

### Inducible Expression of Nr4a1-eGFP

**Figure 9:**
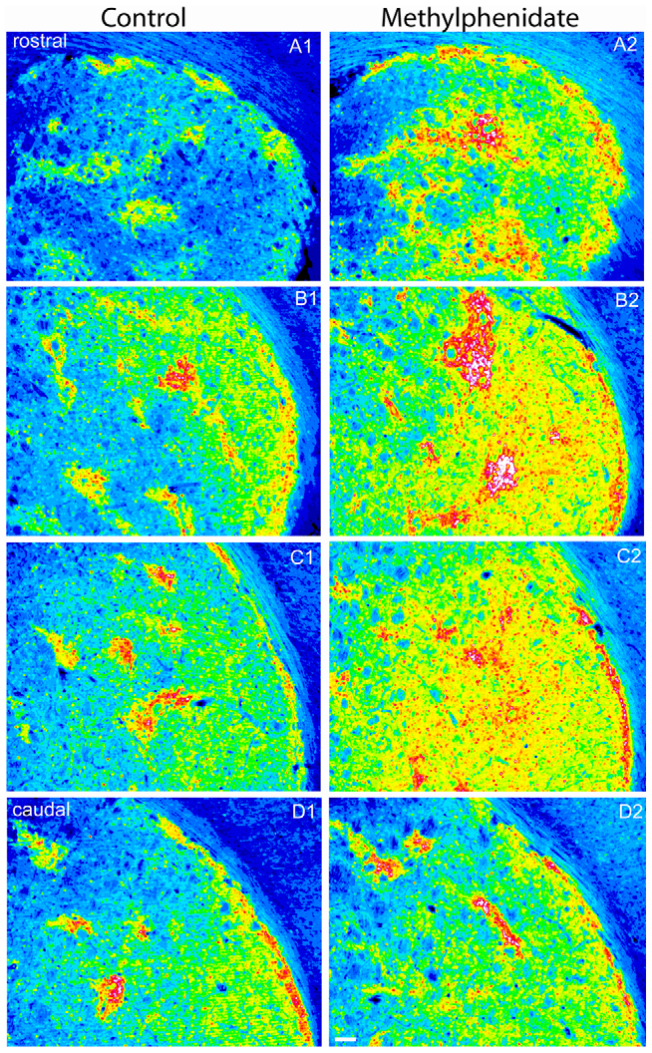
eGFP expression in mice administered 5 mg/kg of methylphenidate. Sections of similar striatal regions are shown for comparison. Images were pseudo-colored using image J (16-bit spectrum). Fluorescence intensity indicates increased levels in the more intense (striosomal, red) regions but also an increase in expression within the matrix (green to yellow shades). Scale bar is 100 µm.

Sensitization to psychostimulants has been suggested to occur through striosomal activation [8], [73] and a single exposure to amphetamine is sufficient to induce immediate-early genes in striosomes [74], [75]. A high dose of methylphenidate (5 mg/kg) was administered to mice by i.p. injection at PN 30 to determine whether eGFP could be induced in a similar fashion to these endogenous immediate-early genes in vivo. Sections from littermate heterozygous mice are presented side by side at 4 levels through the striatum (Fig. 9). MPH increased eGFP levels in the striosomes and the striatal matrix compartment of the DLS (right column) that is consistent with increased locomotor activity. Striatal induction is similar to endogenous Nr4a1 induction [39] as well as Zif/268, Fos and Arc after psychostimulant exposure [74], [75], [76].

**Figure 10:**
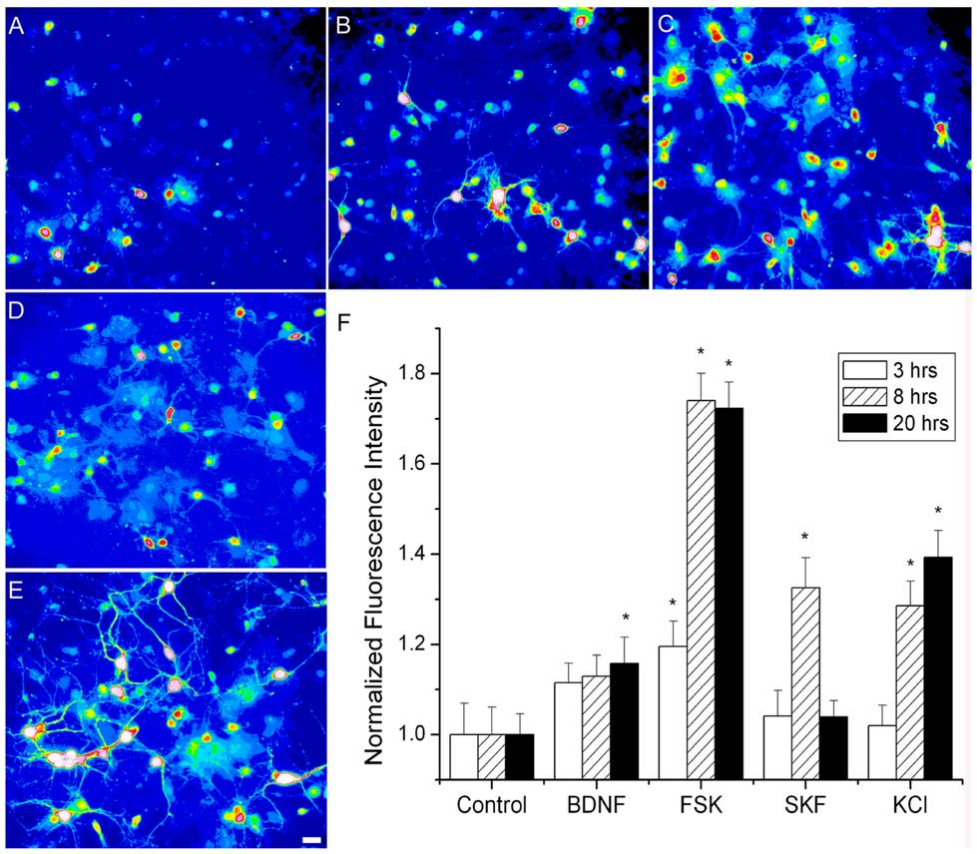
Native fluorescence and fluorometric detection of stimulated eGFP expression in primary cultures of medium spiny neurons stimulated in vitro. Pseudocolored images of control (A), 5 ng/mL BDNF (B), 1 µM forskolin (C), 1 µM SKF-83822 (D) or 30 mM KCl (E) reveal eGFP induction after 20 hrs. eGFP expression was also measured fluorometrically in lysates at 3, 8 and 20 hrs after exposure (F). Native fluorescence is representative of 4 replicate fields taken at the same exposure time (set for linear detection in control cultures). Fluorometry data in F are the mean +/− SE of 16 replicates for each time point and treatment. Data were analyzed by ANOVA (* p<0.05). Scale bar in E (20 µm) applies to all panels.

The apparent activity-dependent regulation and the pattern of expression developmentally suggests a strong association between glutamatergic afferents, dopaminergic afferents, TrkB, ERK and Nr4a1-eGFP expression. However, the lack of colocalization of pCREB and eGFP implicates another level of Nr4a1 regulation in the developing striatum. To determine potential factors regulating the Nr4a1 promoter, MSNs were cultured and treated in vitro with BDNF, forskolin, the Drd1 agonist SKF-83822 or 30 mM KCl for 3–20 hrs. Fluorescence was measured in a microplate fluorometer and examined microscopically (Fig. 10). Cultures contained contaminating glia and large, pyramidal-like cells but MSNs were readily identifiable by morphology and the presence of spines. Stimuli produced different patterns of native fluorescence in the cultures. Expression was infrequent under control conditions (Fig. 10A) with scattered clusters of brightly fluorescent cells visible. BDNF increased fluorescence, filling the cell bodies and making processes of MSNs and contaminating large pyramidal-like cells visible when imaged at the same exposure time as control cells (Fig. 10B). Forskolin increased fluorescence in neuronal-like cells but also induced low level eGFP in contaminating glial cells. Significant differences in eGFP levels were not observed in SKF-83822-treated cells at 20 hrs (Fig. 10D). High extracellular potassium increased eGFP in neurons, with eGFP filling the processes (Fig. 10E). A time course for eGFP expression in response to stimulation measured in lysates is presented in Fig. 10F. Only forskolin stimulation was sufficient to increase eGFP levels after 3 hrs, reaching a maximum increase of 72%. eGFP levels were increased by 8 hrs with forskolin, SKF-83822 and KCl stimulation but 20 hrs was required to detect a 16% increase with BDNF-stimulated eGFP induction. Levels increased or reached a plateau over time with forskolin and KCl treatment (40%) while SKF-83822 induction (32%) was transient.

**Figure 11:**
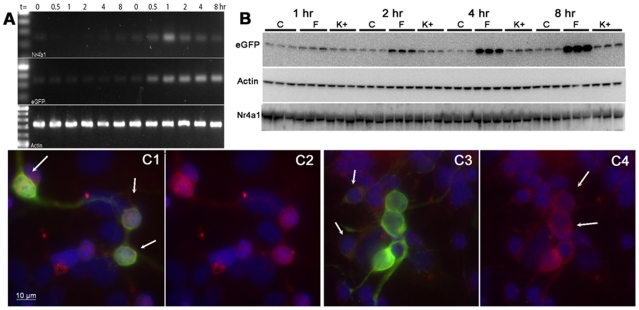
Time course for the in vitro induction of native Nr4a1 mRNA and protein compared to Nr4a1 promoter-driven eGFP expression in MSNs. Semi-quantitative PCR detection of Nr4a1, eGFP and actin mRNA after exposure to 30 mM KCl for 30 min to 8 hrs is shown in A. Western blot detection of eGFP, Nr4a1 and actin induced by 30 mM KCl or forskolin (1 hr to 8 hr) is shown in panel B. Immunofluorescent detection Nr4a1 (red) and eGFP in DAPI stained MSN cultures is shown in C1–C4 after 2 hrs of treatment with 30 mM KCl (C1, C2) or 8 hrs (C3, C4). Arrows in C1 indicate nuclear expression in brightly fluorescent cells after 2 hrs of exposure to 30 mM KCl. DAPI and Nr4a1 are shown together in C2 for this time point. Cells treated for 8 hrs with 30 mM KCl are shown in C3,C4. Arrows (C3) indicate cells with perinuclear eGFP and Nr4a1 immunoreactivity. The DAPI and Nr4a1 channels are shown merged in C4. Arrows (C4) indicate the absence of Nr4a1 immunoreactivity in the nuclei of brightly eGFP-expressing cells at 8 hrs. Scale bar in C1 (10 µm) applies to all images.

Induction and translation of eGFP sufficient to be detected fluorometrically in vitro required 3–20 hrs depending on the stimulus, however the half life of Nr4a1 is 2–4 hrs [31], [36], [63]. The discrepancy between eGFP expression and native Nr4a1 expression in the mature brain was also noted above with immunofluorescence (Fig. 5). To address these differences we performed time course experiments for Nr4a1 and eGFP mRNA and protein expression (Fig. 11). Cultures were stimulated with 30 mM KCl for 0.5 to 8 hrs and semi-quantitative RT-PCR used to detect mRNA (Fig. 11A). Nr4a1 mRNA peaked at 1 hour while eGFP mRNA remained elevated through the 8 hour time point. eGFP protein levels detected by Western blot paralleled eGFP mRNA (Fig. 11B) but there was no correlation between Nr4a1 protein levels and Nr4a1 mRNA in lysates. This could result from differential responses in different MSN subtypes or other post-transcriptional factors; therefore, we examined the expression of Nr4a1 by immunofluorescence. MSN cultures are heterogeneous with respect to direct vs. indirect phenotype but also in the basal level of Nr4a1-eGFP expression with clusters of brightly fluorescent cells (Fig. 11 C). Nr4a1 was detected at 2 hrs in the nuclei of the brighter cell population (red channel, Fig. 11 C1, C2) but was absent from the nuclei of these cells at 8 hrs (Fig. 11 C3, C4, nuclei indicated by the arrows in C4). Interestingly, at 8 hrs both Nr4a1 and eGFP immunoreactivity could be observed in a perinuclear pool in cells with little other cellular eGFP expression (Fig. 11 C3, arrows). This pattern is consistent with ER localization and suggests a different rate of transactivation or translation in this population of cells. It should also be noted that as with immunostaining in the mature brain (Fig. 5), cultures had diffuse staining consistent with mitochondrial localization of Nr4a1 [34], [36].

**Figure 12:**
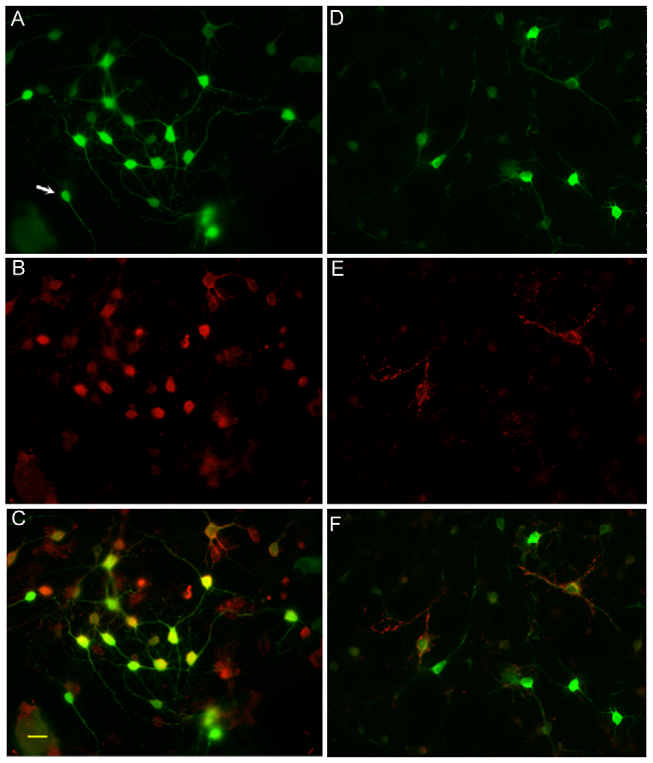
Immunodetection of Drd1, met-enkephalin and eGFP in cultured MSNs. eGFP (A) and Drd1 immunoreactivity (B) are shown merged in panel C. eGFP (D) and met-enkephalin (E) immunoreactivity was detected together in ∼15% of cells expressing high levels of eGFP (merge in F). Cells were treated for 12 hr with 30 mM KCl prior to immunostaining. Scale bar in C (20 µm) and applies to all images.

To further characterize the MSN subtypes with high eGFP expression, cultures treated with 30 mM KCl were stained for Drd1 and met-enkephalin immunoreactivity to determine the cell type with robust activation of the Nr4a1 promoter (Fig. 12). We tested multiple sources of other markers but unfortunately only the Drd1 and met-enkephalin antibodies proved reliable. Intense expression of eGFP (Fig. 12A) was frequently seen in clusters of Drd1+ cells (Fig. 12B). Approximately half of the Drd1+ cells did not express high levels of eGFP (merged image, Fig. 12C). Cells expressing eGFP but no Drd1 were infrequent (∼5%) and often small and bipolar in morphology (arrow, Fig. 12A). eGFP (Fig. 12D) colocalized with punctate/vesicular met-enkephalin immunoreactivity in ∼15% of neurons (Fig. 12E, merge in F).

## Discussion

Expression of eGFP from the Nr4a1 promoter is observed throughout the body and nervous system (Figs. S1 and S2). Expression is not ubiquitous in the brain but within distinct neuronal populations in each brain region, particularly in the striatum, NAc and striatal-like regions of the amygdaloid complex. We characterized expression in the mature animal and explored the utility of this strain for experiments examining striosome-matrix interactions, ontogeny and differential plasticity. Double label immunofluorescence with classical striosome and matrix markers confirmed higher expression in the adult striosomes and concordant expression in developing striosomes. Expression in vivo was temporally and spatially associated with the developing corticostriatal and dopaminergic pathways. Nr4a1-eGFP expression overlaps spatially with dopaminergic innervation, Drd1 expression, TrkB expression and ERK phosphorylation in the neonatal striatum. In vivo exposure to methylphenidate (Fig. 9) and in vitro stimulation increases eGFP expression (Figs. 10, 11, 12) indicating activity-dependent regulation. Thus the Nr4a1 strain is not only an architectural marker of striosomal neurons but also as an inducible reporter of MSN activity.

The Nr4a1 mouse has distinct expression in striosomal neurons under basal conditions and Nr4a1 is enriched in direct pathway neurons (Figs. 1, 6 and 12). Lobo and colleagues [37] identified Nr4a1 as a differentially expressed (1.5 fold) mRNA species in array studies of FACS sorted Drd1- and Drd2-eGFP neurons. These data agree with the difference in eGFP levels between direct and indirect pathway neurons but low eGFP expression in indirect pathway neurons in the Nr4a1-eGFP mouse suggest more heterogeneity than simply direct and indirect. This is further complicated by the observation that Nr41 is expressed in Drd2/enkephalin expressing neurons (Fig. 12 and [33], [37], [39]). Backman and Morales [39] estimated that 25% of met-enkephalin+ neurons expressed Nr4a1 after amphetamine exposure, which is slightly higher than the 15% co-expression we observed. The neurons in the matrix in Fig. 5 that do not express eGFP but have endogenous Nr4a1 immunoreactivity and the matrix cells expressing eGFP after MPH exposure may be these Nr4a1-expressing indirect pathway neurons but these disparities also point to a complex mode of regulation that differs between the striosome and matrix, as has been suggested to exist for phosphorylation of CREB [77].

Differential (high and low) eGFP expression in the striosome and matrix of primarily Drd1-expressing cells under basal conditions and in the developing mouse reveals both a phenotypic and an anatomical division of direct pathway neurons, with striosomal direct pathway neurons having the greatest fluorescence. It has been suggested that the MSN population is composed of at least 5 putative subpopulations. These include direct pathway striosome neurons, indirect pathway striosome neurons, direct pathway matrix neurons and indirect pathway matrix neurons [1]. Cells co-expressing Drd1 and Drd2 or enkephalin and substance P [2], [16], [78] may represent the fifth type. Nr4a1-eGFP expression is most consistent with expression in direct pathway striosomal neurons but a small proportion of met-enkephalin expressing cells also express eGFP in vitro. Nr4a1-eGFP expression and anatomical localization will be an additional tool to determine if functional differences exist between neuronal subtypes in each compartment.

The development of Nr4a1 expressing neurons parallels the development of Drd1 and essentially replicates previous studies on striatal development using other techniques. Developing striosomes contain TrkB, pERK, Drd1 and are heavily innervated by dopaminergic fibers. Surprisingly, CREB phosphorylation did not correlate with eGFP expression in the developing striatum and cAMP is known to induce endogenous Nr4a1 [28]. CREB shows divergent regulation in the striosome vs. matrix. Sustained Drd1 activation increased striosomal CREB phosphorylation while sustained calcium channel activation increased phosphorylation in the matrix in organotypic cultures indicating divergent regulation [77], [79], [80]. The transient nature of pCREB phosphorylation and differential regulation do not exclude CREB as a regulator of Nr4a1-eGFP expression, as transcription factors work in a temporally concerted fashion to induce gene transcription. The lack of association between pCREB and eGFP expression postnatally (Fig. 9) and induction of Nr4a1-eGFP by calcium and forskolin in vitro (Figs. 10 and 11) implies that Nr4a1-eGFP expression may be actively repressed in matrix cells in vivo. Induction by forskolin in vitro suggests that the factors mediating repression can be over-ridden by a strong stimulus or may not be present in vitro (i.e. afferents).

Nr4a1 promoter-driven eGFP expression does not recapitulate endogenous Nr4a1 protein expression in the mature mouse brain under basal conditions (Fig. 5 C,D) or after induction in vitro (Fig. 11) in MSN cultures when measured by Western blot in total lysates. This is in contrast to colocalization in the developing brain (Fig. 5 A,B). The lack of colocalization of eGFP and Nr4a1 in the mature brain is likely due to numerous factors. First, the half life of the two mRNA species is different (Fig. 11). The endogenous mRNA is subject to 3′ regulation that is not present in the BAC construct and the mRNA for eGFP is further stabilized by a viral polyadenylation signal [64]. Second, the half lives of eGFP and Nr4a1 protein also differ (26 hrs [81] vs. 2–4 hrs [36], [63]). Finally, there appears to be a stable pool of Nr4a1 that does not associate with the nucleus (Figs. 5 and 11 and unpublished observations) and appears as a background at low power. However, when cultures were immunostained and examined microscopically we noted that the time course of nuclear localization in the brightly fluorescent cells was rapid, leaving the nucleus by 8 hrs. In contrast, perinuclear immunoreactivity for both Nr4a1 and eGFP was observed after 8 hrs in the dimly fluorescent cells, presumably corresponding to the matrix neurons. These data suggest that the strength or speed of induction may differ between striosomal cells and matrix cells. Consistent with this, Tian and colleagues [33] reported Nr4a1 induction in Drd2-expressing cells after 24 hrs of exposure to high KCl, while Heiman and colleagues [38] did not identify Nr4a1 as a differentially regulated protein in direct vs. indirect pathway neurons by translational profiling of sorted Drd1 and Drd2 cells. Combined, these data indicate that the difference between eGFP levels and native Nr4a1 levels reflect a dissociation in mRNA and protein regulation that differs between MSN populations.

Phosphorylated MEF2 represses Nr4a1 transcription and the calcium-activated phosphatase calcineurin is responsible for dephosphorylation of MEF2 [32], [82]. Striatopallidal neurons show induction of endogenous Nr4a1 in response to calcium channel activation in corticostriatal cocultures [33] and this is consistent with low expression in the matrix under basal conditions that is derepressed by synaptic activity through calcineurin [31], [32], [33], [82]. Calcineurin is high in developing striosomes [83] therefore decreased striosomal pCREB [84] and increased Nr4a1-eGFP may result through the same pathway, activity-dependent activation of calcineurin. These data do not exclude a role for cAMP and low levels of pCREB were present in the striosomes but implicate an activity-dependent MEF-like pathway as a gatekeeper. Divergent expression of endogenous Nr4a1 and the eGFP reporter demonstrates complex regulation at the levels of translation, localization and degradation that should be considered when following eGFP reporter expression.

Induction of Nr4a1 is associated with MSN synaptic remodeling [33] and high levels may therefore mark neurons undergoing plasticity in vivo after drug exposure or after various learning paradigms. Combined with fluorescence activated cell sorting, Nr4a1-eGFP mice can be used to characterize the development and function of a subset of direct pathway neurons that are enriched and activated preferentially in striosomes. Nr4a1-eGFP expression can be used to monitor the convergence of afferent pathways in vitro and in genetic mouse models such as cortex-specific deletion of BDNF or deletion of TH. Activation of prefrontal circuits that preferentially project to the striosomes during behavioral paradigms that switch from goal directed to habitual behaviors or during reversal learning could lead to differential eGFP expression in striosome vs. matrix neurons. Optical monitoring of striosomal neurons in vivo is also feasible with this strain. In addition, variable expression between animals in the striatal-like regions of the amygdala indicates that the Nr4a1-eGFP strain might be useful for identification of circuits involved in anxiety-like behavior. Three-dimensional serial reconstruction technologies have been developed to document complete pathways and connections. Adding the dimension of fluorescence intensity to the reconstruction algorithms would be a phenomenal advance in elucidating complex pathways regulating mouse behavior.

## Methods

### Mice

All animal procedures were carried out in accordance with the NIH Guide for the Care and Use of Laboratory Animals and were approved by the NIAAA animal care and use committee under protocol LIN-DL-21. All efforts were made to minimize suffering of animals. Transgenic Nr4a1 (Tg(Nr4a1-EGFP139Gsat)), Drd1 (Tg(Drd1-EGFP-X60Gsat)) and Drd2 (Tg(Drd2-EGFP-S118Gsat)) were obtained from GENSAT and bred to Swiss-Webster mice (NIH). Experiments were performed on heterozygotes. Mice were genotyped at birth by fluorescence of the thymus, tails and ears using a hand held UV source.

### Staining Procedures

#### Fixation

Adult mice were deeply anesthetized with isofluorane and transcardially perfused with 4% formaldehyde in phosphate-buffered saline (PBS). Neonatal mice were euthanized by rapid decapitation and brains were fixed in 4% formaldehyde for 24 hrs at 4C. Perfusion-fixed brains were post-fixed overnight in 4% formaldehyde. Neonatal brains were sectioned at 50 µm and adult brains were sectioned at 40 or 50 µm as indicated using a vibratome.

#### Immunofluorescence

Sections were blocked with 5% bovine serum albumin in Phosphate-Buffered Saline, 0.02% Triton X-100 (PBS-T) for 4 hrs and then incubated in primary antibodies overnight at 4C. Antibodies used were as follows: chicken anti-GFP (Abcam, 1∶2000), rabbit anti-calbindin (Millipore, 1∶200), rabbit anti-calretinin (Millipore, 1∶1000), rabbit anti-mu-opioid receptor (Immunostar, 1∶5000), rat anti-Drd1 (Sigma, 1∶250), rabbit anti-Nur77 (Santa Cruz, 1∶50) or chicken anti-TH (Abcam, 1∶2000). Rabbit anti-GFP (Millipore, 1∶200) was used for double labeling with TH. Following 3 washes in PBS-T, sections were incubated overnight in secondary antibodies. Alexa 488 goat anti-chicken (1∶2000) and Alexa 568 goat anti-rabbit (1∶1000) or Alexa 555 goat anti-rat (Drd1, 1∶1000) were used in experiments except when in combination with chicken anti-TH where Alexa 568 goat anti-chicken (1∶1000) and Alexa 488 goat anti-rabbit (1∶200) were used (Invitrogen). All antibodies were well characterized and titrated to determine the optimal concentration. Control sections were treated as described with omission of the primary antiserum. Immunofluorescence on cell cultures was performed as described above on cells plated in 8-well chamber slides, except the incubation times were shortened to 1 hr.

#### Counterstaining

Sections were then counterstained with DAPI (Invitrogen) in PBS (0.25 ng/mL), washed 3 times in PBS-T. Alexa-568 IB_4_ isolectin binding (1 µg/mL in PBS-T, Invitrogen) was used for detection of the blood vessels (Fig. S2). Fifty micron sections were incubated overnight and washed three times as described above. Sections were mounted with Fluoromount G (Electron Microscopy Sciences) and sealed with nail polish.

#### Cell Culture

Primary cultures of MSNs were prepared from mice at 0–2 days of age. Striata were dissected on ice, minced and dissociated with papain prepared in MEM (Worthington, 20 U/mL) for 30 min at 30C. Cells were centrifuged at 600 g and resuspended in MEM supplemented with glucose to 25 mM, 2% B-27 supplement, 1 mM sodium pyruvate, 1 mM Glutamax, 100 U/mL penicillin, 100 µg/mL streptomycin, 5% fetal bovine serum and 0.5 µl/mL Mito Serum Extender. All cell culture media and supplements were from Invitrogen except for the fetal bovine serum (Hyclone) and Mito Serum Extender (BD Biosciences). Cells were grown on poly D-lysine coated culture plates (Costar) or 8-well chamber slides (Electron Microscopy Sciences). After 2 days in vitro, medium was changed to medium prepared as above but without serum. Cells were maintained at 37C, 5% CO_2_ in a humidified incubator. Half of the medium was replaced every 3-4 days until assay at 7–10 DIV.

#### In Vitro EGFP Measurements

eGFP expression was analyzed as previously described [85]. Cells were treated with either 5 ng/mL BDNF, 1 µM forskolin (Tocris), 1 µM SKF-83822. or 25 mM KCl (30 mM final) diluted into cell culture medium without serum or Mito Serum Extender. Control wells for forskolin and SKF-83822 contained a final concentration of 0.01% DMSO, although this did not change eGFP expression and control values were collapsed into a single group for analysis. After 3–20 hrs of incubation wells were lysed with 75 µL 0.1% Triton X-100 in PBS (with calcium and magnesium) and homogenized using an ELISA plate shaker. Fifty µL of homogenate was transferred to a black half-well plate and fluorescence was measured using a TECAN microplate fluorometer with the FITC filter set with the background (lysis buffer alone) subtracted. Data were normalized to the average of the control wells on each plate for each time point since B-27 and 30 mM glucose (control) increased eGFP expression.

#### Electrophoresis and Western Blot

Nr4a1 was induced by media change even into formulations containing no additional growth factors so acute induction experiments were performed by treating with concentrated stocks at 5X in MEM with 5 mM glucose, which did not result in significant Nr4a1 induction over the 30 min to 8 hr time course. Cells were stimulated with 30 mM KCl or 5 µM forskolin (final) and lysed with 1% Triton X-100 containing protease and phosphatase inhibitors (Sigma Mammalian Protease Inhibitor Cocktail and Phosphatase Inhibitor Cocktails 1 and 2). Lysates were sonicated, diluted with 2X Laemmli buffer containing 3% (v/v final) 2-mercaptoethanol [86] and heated to 95C for 5 min. Proteins were resolved on 11% SDS-polyacrylamide gels and transferred at 50 mA for 16 hrs onto PVDF membranes according to the method of Towbin [87]. Blots were washed with Tris-Buffered Saline containing 0.05% Tween-20 (TTBS) and blocked with 5% fat-free powdered milk in TTBS. Rabbit anti-Nr4a1 (Santa Cruz, 1∶5000), mouse anti-GFP (Neuromab, 1∶2000) or rabbit anti-B actin (Cell Signaling, 1∶1000) were diluted in TTBS and incubated overnight at 4C. Blots were washed and incubated for 1 hr with HRP-conjugated anti-rabbit (Pierce, 1∶20,000) or HRP-conjugated anti-mouse (Bio-Rad, 1∶1000). Following a final series of washes, immunoreactivity was visualized using chemiluminescence (Pierce Super Signal Pico West) and imaged using a Kodak Image Station. The same blots were sequentially reprobed with each antibody.

#### Semi-quantitative RT-PCR

Cells were cultured in 48 well culture dishes and treated for 0–8 hrs with 30 mM KCl as described above for protein expression. Cells were lysed in the culture dish wells by addition of 300 µL of buffer RTL, a component of the RNeasy kit from Qiagen. Total RNA was isolated using this kit and 10 µL of the resulting total RNA was reverse transcribed using the QuantiTect Reverse Transcription kit (Qiagen). The 20 µL final reaction product was diluted to 120 µL and 2 µL of cDNA was transferred to one of 3 reaction tubes for amplification of Nr4a1, eGFP or actin in 50 µL reactions using 2X GoTaq Hot Start Polymerase Green Master Mix (Promega). Primer pairs (300 nM final reaction concentration) were (5′ to 3′): mouse Nr4a1 (for GTCCGCACCTGTGAGGGCTGC, rev GGTAGGGGAGGCATCTGGAGGC): eGFP (for CCTACGGCGTGCAGTGCTTCAGC, rev CGGCGAGCTGCACGCTGCCGTCCTC) and for mouse B-actin (for CTGAGGAGCACCCTGTGCTGC, rev CCAGGATGGAGCCACCGATCC). Seven µL aliquots of product were taken after 22, 27, and 37 cycles and 5 µL of the aliquot was analyzed by 1.8% agarose gel electrophoresis. Bands were visualized using Syber-Safe (Invitrogen) and fluorescence captured using a Carestream Gel Logic 112 Imager.

#### Methylphenidate Exposure

Heterozygous mice were divided by litter and gender so that litter mates and cage mates of the same gender were used for comparison at each time point. Mice were injected at PN30 with 5 mg/kg methylphenidate in normal saline or vehicle and sacrificed 12 hrs later. Brains were block fixed in cold 4% formaldehyde for 24 hrs and processed within 1 week. Every 3rd section through the striatum was imaged and landmarks were used to insure imaging of similar striatal regions. eGFP antibody staining (above) was used in these experiments because of poor eGFP stability in stored tissue and the time required to section and image the brains.

#### Microscopy and Quantification of Immunofluorescence

Sections were imaged with a Zeiss Axiovert epifluorescence microscope or a Zeiss Lumar stereo-microscope equipped with standard DAPI, eGFP and Cy3 filter sets (Chroma). Images were captured with an Axiocam MR using the Axiovision software. For some figures binary images were converted to pseudo-colored images using Image J (Scion Corp.) for presentation as indicated in the figure legends using the 16-bit spectrum lookup tables. Images were collected using the same exposure times and background subtractions (when used) were linear and the same for all sections within each experiment when quantitative comparisons were made.

## Supporting Information

Figure S1 **Nr4a1-eGFP expression within the central nervous system in the adult mouse.** Diffuse expression was observed throughout the brain in fibers and somata. Panels are labeled with the approximate location relative to Bregma according to the Allen Brain Atlas coordinates for an adult C57/Bl6J mouse and are therefore approximate. The predominant structure in each panel is indicated. Abbreviations: Olf, oflactory; PrL, prelimbic cortex, Pir, piriform cortex; M1/M2, motor cortex 1/2; Hab, habenula; MeA, medial amygdala; Hyp, hypothalamus; Au Cx, auditory cortex; LGN, lateral geniculate nucleus; HP, hippocampus; vHP, ventral hippocampus; PAG, periaquiductal grey; Coll, colliculi; CB, cerebellum. Panels are of the left hemisphere except for medial regions (B3, C1, E1).
(TIF)

Figure S2 **Gross survey of peripheral expression of Nr4a1-eGFP.** eGFP fluorescence was detected in the intestine (A), muscle and spinal ganglia (B), heart (C), spleen (D), testes (E, higher magnification, E′) and lung (F). eGFP in the liver (G) was primarily associated with the vasculature (G′ stained with IB_4_ isolectin). Expression in the kidney (H) was also associated with the vasculature (higher power shown in I, Alexa-568 IB_4_ isolectin binding is shown in I′). Images were taken with a Zeiss Lumar stereomicroscope (A, B, E, F, H) or a Zeiss Axiovert epifluorescence microscope (D, E′, G, G′, I, I′). Scale bars are present in each panel.
(TIF)
