# Correction: Nr4a1-eGFP Is a Marker of Striosome-Matrix Architecture, Development and Activity in the Extended Striatum

**DOI:** 10.1371/annotation/83671a73-38e3-4ba4-b016-81264138a0ac

**Published:** 2011-02-17

**Authors:** Margaret I. Davis, Henry L. Puhl

There is an error in the first sentence of the funding section. The sentence should read: "This work was supported by the National Institutes of Health, National Institute on Alcohol Abuse and Alcoholism (NIAAA) Division of Intramural Clinical and Biomedical Research."

