# Supplementary figures and images for: Nr4a1-eGFP Is a Marker of Striosome-Matrix Architecture, Development and Activity in the Extended Striatum
Source: PLoS One. 2011 Jan 28;6(1):e16619. doi: 10.1371/journal.pone.0016619 (PMC3030604; doi:10.1371/journal.pone.0016619)

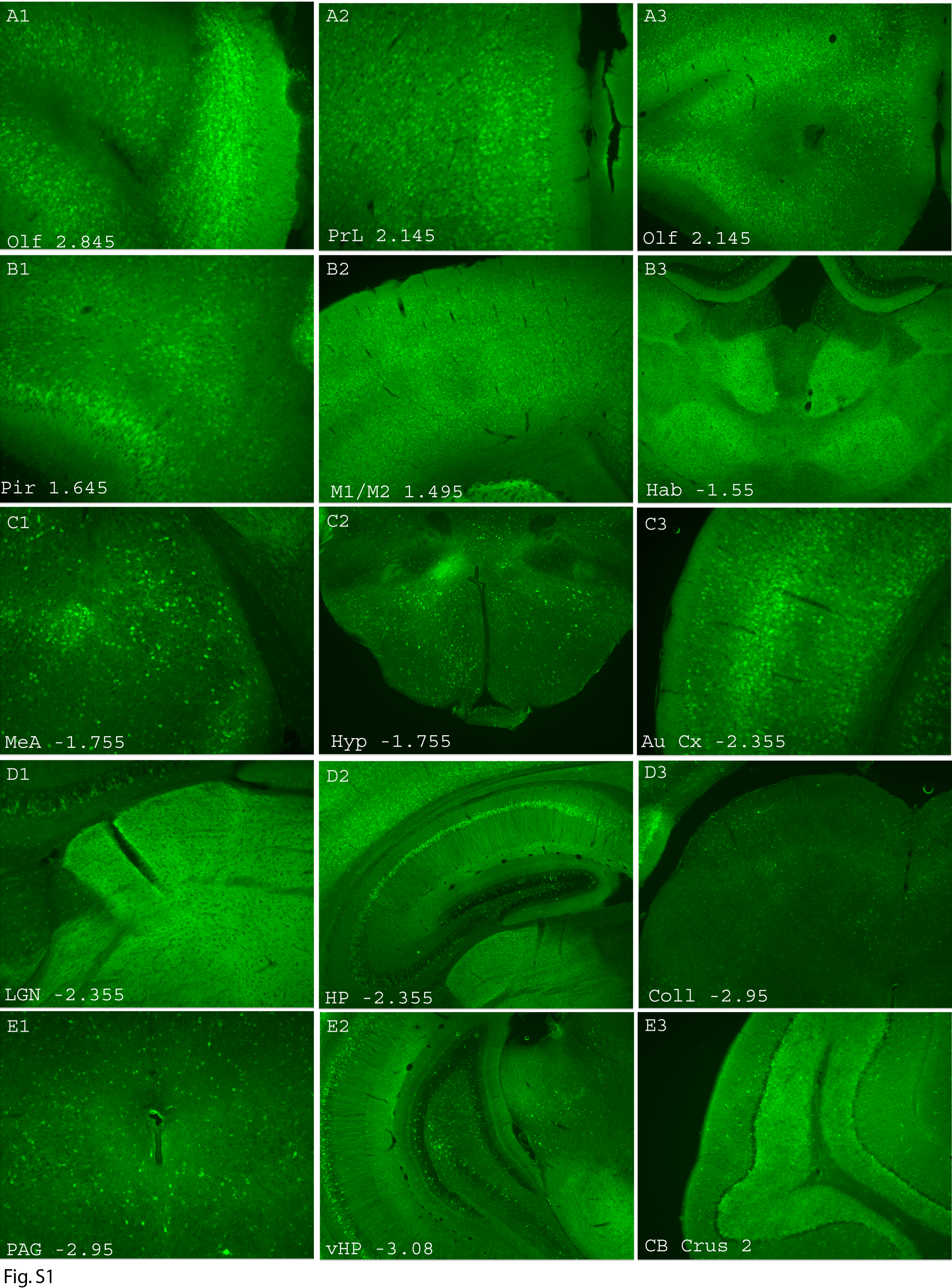

Supplement: Figure S1 — Nr4a1-eGFP expression within the central nervous system in the adult mouse. Diffuse expression was observed throughout the brain in fibers and somata. Panels are labeled with the approximate location relative to Bregma according to the Allen Brain Atlas coordinates for an adult C57/Bl6J mouse and are therefore approximate. The predominant structure in each panel is indicated. Abbreviations: Olf, oflactory; PrL, prelimbic cortex, Pir, piriform cortex; M1/M2, motor cortex 1/2; Hab, habenula; MeA, medial amygdala; Hyp, hypothalamus; Au Cx, auditory cortex; LGN, lateral geniculate nucleus; HP, hippocampus; vHP, ventral hippocampus; PAG, periaquiductal grey; Coll, colliculi; CB, cerebellum. Panels are of the left hemisphere except for medial regions (B3, C1, E1). (TIF) [file pone.0016619.s001.tif]

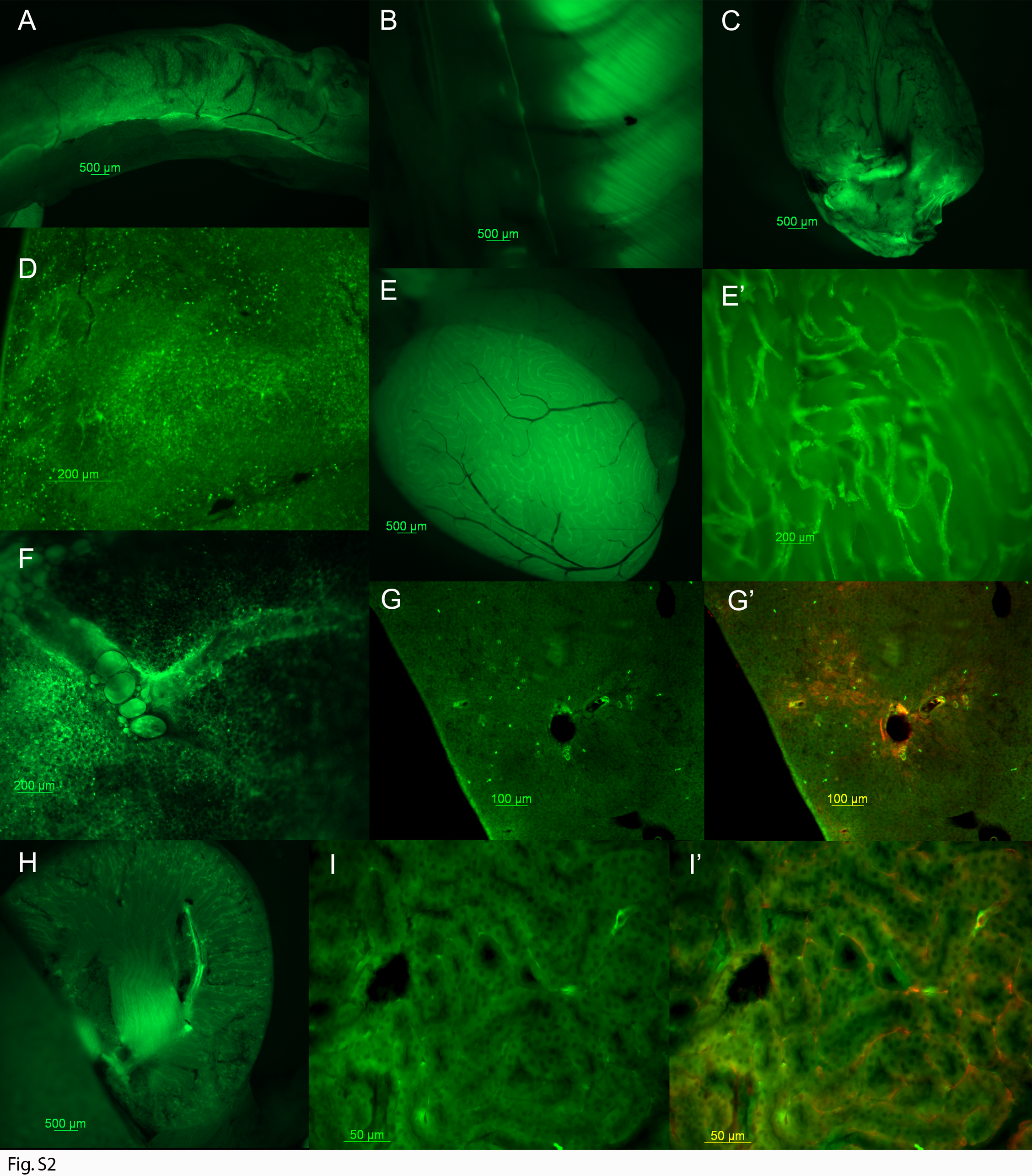

Supplement: Figure S2 — Gross survey of peripheral expression of Nr4a1-eGFP. eGFP fluorescence was detected in the intestine (A), muscle and spinal ganglia (B), heart (C), spleen (D), testes (E, higher magnification, E′) and lung (F). eGFP in the liver (G) was primarily associated with the vasculature (G′ stained with IB4 isolectin). Expression in the kidney (H) was also associated with the vasculature (higher power shown in I, Alexa-568 IB4 isolectin binding is shown in I′). Images were taken with a Zeiss Lumar stereomicroscope (A, B, E, F, H) or a Zeiss Axiovert epifluorescence microscope (D, E′, G, G′, I, I′). Scale bars are present in each panel. (TIF) [file pone.0016619.s002.tif]
